# Resistance to Snow Mold as a Target Trait for Rye Breeding

**DOI:** 10.3390/plants11192516

**Published:** 2022-09-26

**Authors:** Mira Ponomareva, Vladimir Gorshkov, Sergey Ponomarev, Gulnaz Mannapova, Danil Askhadullin, Damir Askhadullin, Olga Gogoleva, Azat Meshcherov, Viktor Korzun

**Affiliations:** 1 Federal Research Center “Kazan Scientific Center of the Russian Academy of Sciences”, 420111 Kazan, Russia; 2 KWS SAAT SE & Co. KGaA, Grimsehlstr. 31, 37555 Einbeck, Germany

**Keywords:** rye, snow mold, resistant sources, crop genetic resources, plant infectious diseases, breeding

## Abstract

Winter rye is a versatile crop widely used for food and industry. Although rye is resistant to abiotic stressors and many phytopathogens, it is severely damaged by pink snow mold (SM)—a progressive disease caused by the psychrotolerant fungus *Microdochium nivale* under the snow cover or during prolonged periods of wet and cool conditions. Due to little use of the SM resistance sources in contemporary breeding, varieties with at least moderate resistance to SM are limited. Our study aimed to integrate field assessment under natural conditions and an artificially enriched infection background with laboratory techniques for testing rye accessions and selecting SM resistant sources for applied breeding programs and genetic research. We revealed valuable sources of SM resistance and split rye accessions, according to the level of the genetic divergence of the SM resistance phenotype. This allowed us to select the most distinct donors of the SM resistance, for their use as parental forms, to include novel variability sources in the breeding program for achieving high genetic variability, as well as enhanced and durable SM resistance, in progeny. The rye accessions analyzed here, and the suggested options for their use in breeding, are valuable tools for rye breeding.

## 1. Introduction

Winter (fall) rye (*Secale cereale* L.) is a relatively recently domesticated cross-pollinated cereal species belonging to one of the three taxa of the *Secale* genus of the *Poaceae (Gramineae)* family [1]. Rye is a versatile crop used for bread making, livestock feeding, bioenergy, and alcohol production [2,3,4]. This crop is frequently associated with healthy nutrition trends due to its well-balanced composition (minerals, vitamins, sterols, phenolic compounds, lignans, alkylresorcinols, phosphorous, magnesium, pantothenic acid, and the highest dietary fiber content compared to other cereals) [5,6,7,8,9,10]. Rye-based products possess probiotic activity, reduce the risk of various diseases, and improve lipid metabolism [11,12,13,14,15].

Rye is a very resistant crop that produces a high yield under unfavorable conditions: severe frost, drought, low fertile soils with irregular pH (5.0–7.0) [16]. In addition, rye is much less susceptible to infectious diseases compared to wheat and barley. Therefore, the production costs of rye are lower compared to other cereals due to the lower use of pesticides, insecticides, and fertilizers [17,18]. However, rye is severely damaged by snow mold (SM) disease, which can lead to large yield losses by reducing the number of productive stems, spike yield, and often plant death.

SM, which usually develops under the snow cover, is caused by psychrophilic and psychrotolerant fungi belonging to distant taxa: *Microdochium nivale*, *M. majus*, *Typhula ishikariensis*, *T. incarnata*, and *Sclerotinia borealis*. In the Central and Western regions of Russia, *M. nivale* is a dominant SM pathogen [19,20]. In the Middle Volga region, significant SM-related yield losses occur once every two years, while in the Volga–Vyatka region, they occur almost every year (9 of 10) [21,22]. The SM outbreaks are usually confined to the years with the longest periods of snow cover [21,22]. Herewith, yield losses accounted for up to 40% [23].

The problem with SM is largely related to the small number of SM-resistant genetic resources used in rye breeding. SM has been long considered to be restricted to those areas where the snow cover is maintained for a long period (more than 100 days), such as Russia, Belarus, and Nordic and Baltic countries [24,25,26]. Therefore, no breeding for SM resistance has been carried out in “warmer” countries, despite the fact that *M. nivale*-caused SM gradually “moves” toward “less snowy” territories (Southern parts of Russia, as well as Western and Eastern Europe) where long periods of cool (4–15 °C) and rainy weather compensate for the absence of snow cover, allowing SM progression [20,27,28,29,30]. In Krasnodar territory (south of Russia with little or no snow), *M. nivale*-caused SM exceeds the epiphytotic level 3–5 times per 10 years, causing yield losses of up to 20% [30]. In addition, *M. nivale* has been shown to cause not only SM but also other diseases on cereal crops in the southern parts of Russia [23,31], Poland [32,33,34], Denmark [35], Japan [36], and China [37] throughout the growing season.

SM damage can be minimized by incorporating new germplasm sources into the breeding programs that will make rye more resistant/tolerant to this disease. The identification and transfer of SM resistance genes to new varieties are difficult tasks since this trait is polygenic and strongly influenced by environmental conditions, requiring several years of field observations. Therefore, rigorous laboratory testing is needed to verify the level of resistance to SM pathogens in addition to field observations. Moreover, rye, as well as other cross-pollinated plants, is difficult to handle in breeding work; the inheritance of gene combinations, which can easily be achieved for self-pollinated plants, represents great difficulties in rye. Despite these difficulties, SM resistance is an important task for the breeding of both population and hybrid rye varieties. Genetic resources are a tool that allows breeding to respond to the challenges of climate change and the emergence of new pathogens [38]. Rye genetic resources are not sufficiently used in practical modern rye breeding for resistance [2], and continuous work on their study and involvement in the breeding process is required [39,40]. The reliable genetic markers of SM resistance would facilitate the breeding of SM resistant rye cultivars. However, no SM resistance-related quantitative trait loci (QTLs) were identified in rye to date, and only a few of them were revealed in wheat and triticale [41,42,43,44].

The aim of the present study was to integrate field assessment, under natural field conditions and artificially enriched infection backgrounds, with laboratory techniques for testing rye accessions from genetic collection for SM resistance and selecting SM resistant sources for applied breeding programs and genetic research of rye.

## 2. Results

### 2.1. Phenotypic Variation of SM Resistance in Rye Accessions

**Figure 1:**
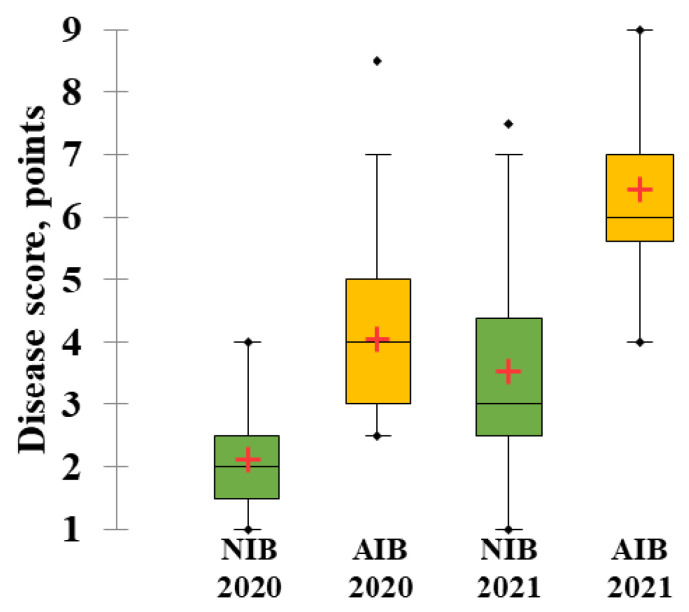
Boxplots showing the variation of the snow mold damage of rye genetic resources, in 2020 and 2021, under a natural infection background (NIB, green boxes) and an artificially-enriched infection background (AIB, yellow boxes). The boxplots show the interquartile range and the whiskers—minimum and maximum values. In each boxplot, the median is shown as a thick horizontal line, and the mean value is a cross. “◆” shows an outlier that is outside the boxplot whiskers and significantly different from the rest of the dataset.

There were 58 rye samples from 13 countries (Supplementary Table S1) tested for SM resistance in the field, under both a natural infection background (NIB) and an artificially-enriched infection background (AIB), during two growing seasons (2019–2020 and 2020–2021). SM damage was significantly greater in 2021 than in 2020 under both infection backgrounds (NIB and AIB) (Figure 1). The average disease score in 2020, under AIB, was 4.04 ± 0.18, and in 2021, it was 6.42 ± 0.15. In both years, SM damage was significantly more severe under AIB compared to NIB (Figure 1).

**Figure 2:**
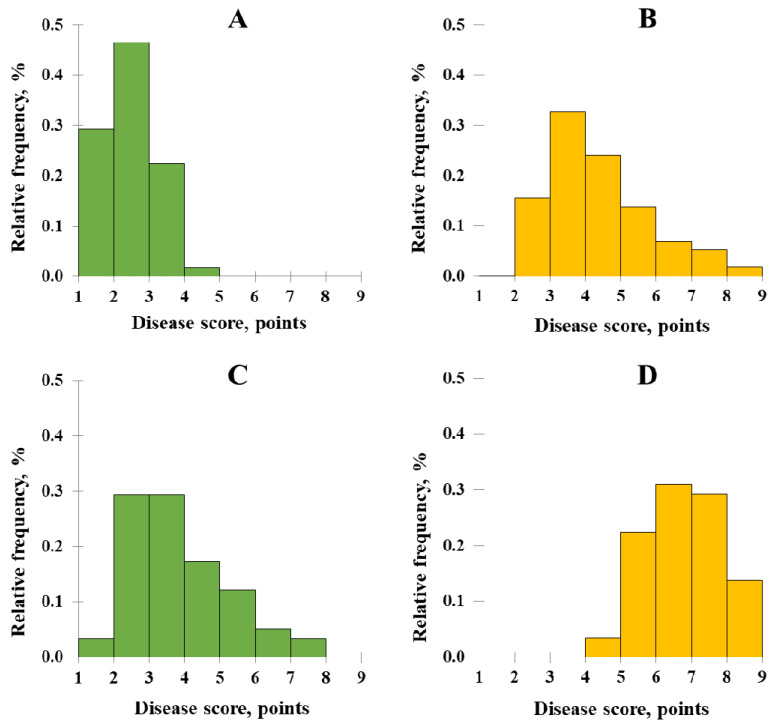
Histograms of the distribution of winter rye samples by the degree of snow mold damage under a natural infection background (NIB, green columns) (**A**,**C**) and an artificially-enriched infection background (AIB, yellow columns) (**B**,**D**) in 2020 (**A**,**B**) and 2021 (**C**,**D**).

In 2020, 29.3% of samples were affected by SM under NIB at the level of disease scores of 1–1.9 points. The highest proportion of accessions (46.6%) had disease scores of 2–2.9 points; 22.4 and 1.7% of samples had disease scores of 3–3.9 and 4–4.9, respectively (Figure 2A). Herewith, under AIB, no samples with disease scores of 1–1.9 were revealed; 15.5, 32.8, 24.1, 13.8, 6.9, 5.2, and 1.7% of samples had disease scores of 2–2.9, 3–3.9, 4–4.9, 5–5.9, 6–6.9, 7–7.9, and 8–8.9 points, respectively (Figure 2B).

In 2021, under NIB, 3.45% of samples were affected by SM at a level of disease scores of 1–1.9 points. The highest proportion of accessions (29.3%) had disease scores of 2–2.9 and 3–3.9 points; 17.2, 12.1, 5.2, and 3.45% of samples had disease scores of 4–4.9, 5–5.9, 6–6.9, and 7–7.9, respectively (Figure 2C). Herein, under AIB, the most pronounced disease progression was registered, with no samples with disease scores of less than 4 (Figure 2D). Among the samples 3.5, 22.4, 31.0, 29.3, and 13.8% had disease scores of 4–4.9, 5–5.9, 6–6.9, 7–7.9, and 8–9, respectively, under AIB.

### 2.2. Differentiation of Rye Accessions by the SM Resistance

Screening of rye accessions of different geographical and ecological origins, under two infection backgrounds, allowed us to identify classes of genotypes with close or identical phenotypes in terms of the SM disease scores (Supplementary Table S2). Of the 58 rye accessions tested under NIB in 2020, most (44) were considered resistant (R) (Table 1). The remaining 14 samples were moderately resistant (MR). In 2021, 19 resistant and 27 moderately resistant cultivars were found. Among the resistant samples (R), 13 samples were of Russian origin, 3 were from Belarus, 1 was from Ukraine, and 2 were from Latvia. For both 2020 and 2021 together, the two way ANOVA (α ≤ 0.05) did not reveal significant differences in disease scores of rye accessions (Supplementary Table S2).

Under AIB, in 2020, 9 samples were considered resistant (1–2.9 points): Ubileinaya 25, Sibirskaya 87, Sinilga, Falenskaya 4, Derzhavinskaya 50, Talovskaya 44, Orlovskaya 9-2 (Russia), Talisman (Belarus), and Pico Uruguay (Uruguay) (Table 1, Supplementary Table S2). In 2021, only Volzhanka 2 and Uralskaya 2 from Russia displayed moderate resistance to SM under AIB. Thirty-one samples were moderately susceptible (MS), and 25 samples were susceptible (S) to SM; no samples were resistant. The susceptible check Conduct had a disease score of 4.5 under NIB and 8.8 under AIB. Considering both 2020 and 2021 together, the disease scores of rye accessions had significant differences (two way ANOVA, α ≤ 0.05, Supplementary Table S2).

**Figure 3:**
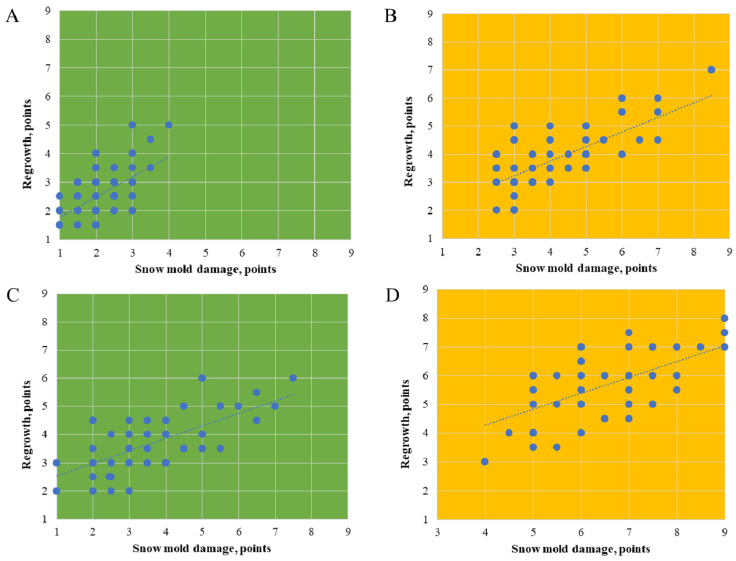
Correlation between snow mold disease scores assessed within a week after the snow melt (evaluation 1) and two weeks after the evaluation 1 (evaluation 2) on 58 rye accessions; evaluation 2 shows plant regrowth ability after the snow mold damage. The evaluations were performed in 2020 (**A**,**B**) and 2021 (**C**,**D**) under a natural infection background (green, **A**,**C**) and an artificially-enriched infection background (yellow, **B**,**D**).

Follow-up observations of the crops two weeks after the first evaluation (during spring regrowth) showed that many accessions had high regeneration ability, formed additional tillering shoots, and recovered well after the SM damage. The correlation of mean SM damage, after the first and the second (during regrowth) evaluation, was significant: r = 0.707 *** and r = 0.551 *** under AIB in 2020 and 2021, respectively, and r = 0.604*** and r = 0.672 *** under NIB in 2020 and 2021, respectively (Figure 3).

### 2.3. Influence of SM on the Agronomic Parameters of Rye

SM severity influenced the agronomic parameters (yield and yield structure) of rye. Under AIB conditions, the values of plant regrowth, plant height, spike length, grain number per spike, and grain weight per spike decreased compared to NIB conditions. The most significant differences were found for such parameters as grain yield and spikelet number per spike (Table 2).

The disease scores of rye accessions determined at both evaluations (one and three weeks after the snow melt) had high negative correlations with grain yield under both NIB and AIB conditions (Table 3). This means that SM progression is an important factor for rye yield losses not only at high (AIB) but also at moderate (NIB) infectious loads. We also found highly positive correlations between spike characteristics (for example, spike length and number of spikelets, or grain number per spike and grain weight); herewith, the correlation coefficient values were greater for AIB than for NIB.

### 2.4. Determination of the SM Resistance by the Detached Leaf Assay (DLA) Test

The detached leaf assay (DLA) test was performed on leaf segments of 34 rye accessions. Detached leaf segments were inoculated with a highly virulent SM causal strain, *M. nivale* F00628. There were three parameters of the infected leaves assessed: disease incidence (DI), leaf infestation (LI), and index susceptibility (IS) (see Section 4).

At the first assessment, the lowest DI was observed for varieties Jan An, Sinilga, and Tatiana (3.3–10.0%), while at the fourth assessment, only the variety Ogonek had a DI below 50% (36.7%) (Supplementary Table S3). The highest DI was registered for the samples Rifle Fall and Krona 2 (86.7% and 100% for the 1st and 2nd assessments, respectively). Among the analyzed samples, one-third were affected by *M. nivale* by 60%, and 90–100% were affected by the third and fourth assessments, respectively. The lowest LI was typical of Ogonek and Sinilga following all four assessments, while the highest LI was observed for Rifle Fall, Krona 2, Zduno, and Parcha (Supplementary Table S3). The two way ANOVA (α ≤ 0.05) revealed significant differences in the levels of DI and LI of rye accessions (Supplementary Table S3).

**Figure 4:**
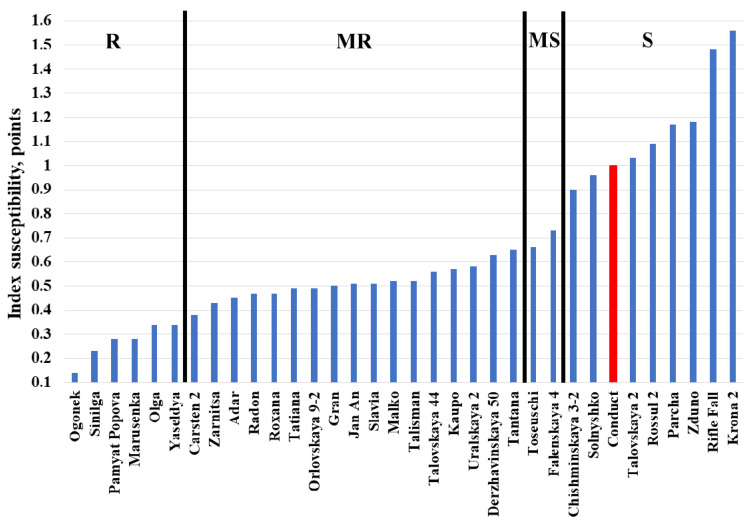
Index susceptibility (IS) showing the level of quantitative resistance of rye accessions to *Microdochium nivale*. The analysis was performed using the detached leaf assay method under laboratory conditions. R—resistant accessions; MR—moderately resistant accessions; MS—moderately susceptible accessions; S—susceptible accessions. The IS of the susceptible check Conduct is shown in the red column.

There were 6 accessions (Ogonek, Sinilga, Pamyat Popova, Marusenka, Olga, Yaseldya) with the lowest IS of 0.10–0.35 (most resistant) (Figure 4). The IS of 50% of the accessions was 0.36–0.65 (moderately resistant). An IS of greater than 0.81 was typical of 9 accessions (susceptible). Although, in general, DI, LI, and IS differentiated individual rye accessions (into different groups: R, MR, MS, or S) in a similar way, some accessions appeared within different groups, in terms of susceptibility to *M. nivale*, following DI, LI, and IS evaluation. For example, Jan An and Talisman had an IS of 0.51 and 0.52, respectively, while their DI was 49 and 81, respectively. This means that, although the level of quantitative resistance was similar for these two accessions, individual leaves of Jan An displayed greater variability in resistance (fewer leaves were affected) than Talisman (for which 81% of leaves were affected). Similarly, the LI for Kaupo and Uralskaya 2 were 21 and 20, respectively, while their DI was 50 and 72, respectively. This means that the level of leaf damage was similar for these two accessions, while individual leaves of Kaupo were more rarely affected by *M. nivale* than the leaves of Uralskaya 2.

### 2.5. Cluster Analysis of Rye Accessions Based on Multiple SM Resistance Assessments

The comparison of the results obtained from field experiments (under AIB and NIB) and the laboratory DLA test revealed that some of the rye accessions displayed similar phenotypes when analyzed using different test systems, while for the other rye varieties, the DLA results did not correspond to field assessments. Among 16 accessions that were moderately resistant under AIB conditions, 12 were also moderately resistant or resistant in the DLA test (Radon, Talisman, Uralskaya 2, Derzhavinskaya 50, Zarnitsa, Gran, Malko, Talovskaya 44, Tantana, Sinilga, Pamyat Popova, Orlovskaya 9-2); the other 4 accessions (Falenskaya 4, Talovskaya 2, Rifle Fall, Rossul 2) were susceptible/moderately susceptible according to the DLA test (Table 4).

Among the accessions, 14 were moderately susceptible under AIB conditions displayed different phenotypes in the DLA test (from resistance to susceptibility). Of the four accessions that were susceptible under AIB, two were also susceptible in the DLA test, and the other two accessions were resistant or moderately resistant in the DLA test (Table 4).

For some accessions, a strong inconsistency between the results of field and laboratory tests was observed. For example, Talovskaya 2, Rifle Fall, and Rossul were among the most resistant genotypes under increased infection background in the field (AIB); however, their leaves under laboratory conditions (DLA) were highly susceptible to *M. nivale* invasion and propagation. In contrast, Marusenka and Jan An were among the most susceptible varieties under field conditions (both NIB and AIB), but the leaves of these varieties in the DLA test did not suffer significantly after infection with *M. nivale* (Table 4). This means that the resistance to *M. nivale* is a “summary” of independent properties of a variety. Each of these properties is likely conferred by distant genes, supporting the polygenic nature of SM resistance. To obtain varieties with a high level of SM resistance, the parental forms should be selected in such a way as to ensure the involvement of genetically diverse resistant traits in the breeding program. To reveal such parental forms, we performed a cluster analysis in order to identify the donors of the most genetically distant traits of SM resistance. The clusterization of rye accessions was based on the results of the analysis of SM damage under all assessed experimental conditions (NIB, AIB, and DLA), and it was carried out based on the dissimilarity principle (Euclidean distance, Ward’s method) in order to differentiate the sources of resistance into different clusters. In turn, the use of accessions from different clusters, as parental forms, will further provide an opportunity to combine genetically distant SM resistance traits in progenies.

**Figure 5:**
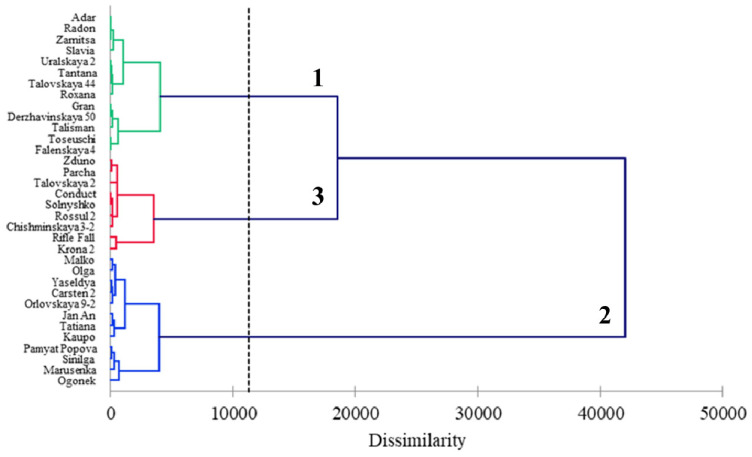
Hierarchical agglomerative clustering of 34 rye accessions, based on the parameters of snow mold damage, assessed under a natural infection background (NIB) and an artificially-enriched infection background (AIB) in the field, as well as under laboratory conditions following *Microdochium nivale* infection using a detached leaf assay. Clustering was performed using Ward’s method; the proximity between objects was measured by the criteria of dissimilarity based on Euclidian distance.

There were 34 rye accessions that were analyzed, under both field and laboratory conditions, and were distributed among three clusters (Table 5, Figure 5). The mean variance within a cluster was 26.5%, while the mean variance between clusters was 73.5%. The first cluster included 13 varieties from Russia, Belarus, Poland, and the United States. Samples from this cluster were, on average, resistant under NIB conditions and moderately resistant under AIB conditions. In the DLA test, these varieties demonstrated moderate resistance. The second cluster consisted of 12 samples from Russia, Belarus, Poland, Latvia, and China. These samples were moderately resistant under NIB conditions, moderately susceptible under AIB conditions, and displayed rather high resistance in the DLA test. The varieties of the third cluster from Russia, Poland, Germany, and Canada were similar to the varieties of the second cluster in terms of their average field SM resistance (disease scores of 2.9 and 5.7 for NIB and AIB, respectively). In the DLA test, the samples from the third cluster demonstrated high susceptibility (Table 5, Figure 5).

Thus, we split the assessed rye genetic resources into three clusters, among which rather high genetic variability of SM resistance traits is expected. The hybridization of the SM resistance sources, attributed to distinct clusters, should yield progeny with increased SM resistance.

## 3. Discussion

In the present study, we conducted a large-scale screening of rye accessions from the collection of the Vavilov All-Russian Institute of Plant Genetic Resources, as well as our own breeding material, for resistance to SM caused by highly virulent *M. nivale* strain F00628. The main emphasis was placed on samples originating from the central part of Russia, where the level of development of this disease is very high, and, therefore, among these samples, the resistant sources are highly expected. Indeed, sources of resistance to SM among Russian rye accessions have been previously reported [21,45]. However, varieties with complete resistance to SM do not exist, and varieties with moderate SM resistance are limited as well. This is due to the fact that a small amount of breeding material with increased SM resistance is used in contemporary breeding. We searched the SM resistance sources in the Tatarstan Republic, where a fairly high level of SM is a typical situation [46], for two years in which the level of natural development of the disease was different: in 2021, the level of SM was significantly higher than in 2020. The variation in SM severity during two years of observation was likely related to different durations of snow cover—102 and 150 days in 2020 and 2021, respectively (Table 6)—which is consistent with the confinement of SM outbreaks to prolonged periods of snow cover [21,22].

SM resistance is a quantitative trait controlled by a few major genes and many individual loci, the products of which have additive effects in conferring resistance [47,48]; here, phenotypes determined by different groups of SM resistance genes are presumably manifested under different conditions. Some quantitative trait loci (QTLs) related to SM resistance have been revealed in winter wheat and triticale [41,42,43,44]. Some of these QTLs were also associated with frost tolerance. To conclude whether these QTLs directly determine SM resistance or contribute to the general fitness of plants during the winter period by conferring increased cold tolerance requires further investigations. In winter rye, the genetic markers of SM resistance have not been identified to date.

The development of a resistance test for winter rye to SM is an important objective of breeding (including hybrid breeding) and is necessary for creating mapping populations for the identification of SM resistance genetic markers [49]. In this regard, for the phenotyping of samples, we used an integrated approach and analyzed them using three complementary test systems: NIB, AIB, and DLA.

The first test system (NIB) allows for assessing the SM resistance at the natural level of the disease, which can vary significantly depending on the weather conditions of a particular year. In some years, the level of SM development can be very low (for example, during cold winters with little snow and a short off-season period) and, therefore, it is not possible to differentiate the level of SM resistance of different accessions. The second test system, based on the determination of the SM resistance at an increased infectious load (AIB), neutralizes this problem and, additionally, makes it possible to check how different rye varieties manifest themselves during epiphytotic years. In our study, an increased infectious load (AIB) caused a 42% yield loss compared to the yield amount under NIB, which corresponds to the losses observed during years of SM outbreaks [23].

The third test system (DLA) allows for assessing the SM resistance of the detached leaves to the invasion of SM fungi and their spread over the surface of the leaf blade under controlled laboratory conditions. In addition, the DLA test is the fastest and most easily reproducible method for evaluating breeding material. We presumed that, within different test systems, the contribution of different resistance genes to the observed resistance will be manifested to different degrees, which is of great importance for the proper selection of parental forms for creating varieties with increased SM resistance.

The results of the determination of the SM resistance level, under field conditions largely depend on the weather conditions of a particular year [50], while the laboratory tests are quite artificial, and their results may not be reproduced in the field. Therefore, testing under both field and laboratory conditions seems to be the most reasonable way of screening the genetic resources for resistance to infectious diseases. In our study, twelve rye accessions (Radon, Talisman, Uralskaya 2, Derzhavinskaya 50, Zarnitsa, Gran, Malko, Talovskaya 44, Tantana, Sinilga, Pamyat Popova, Orlovskaya 9-2) displayed the highest level of SM resistance under both field and laboratory conditions. These accessions can be regarded as valuable sources of SM resistance.

The use of a variety of distinct donors is of particular importance for breeding SM resistance since this is a polygenic trait with low heritability for selection [51]. Herewith, different gene groups control different aspects of SM resistance, whose combination is required for high level and durable resistance. To find the most distinct donors of the SM resistance, we performed a cluster analysis to classify rye accessions according to the level of the genetic divergence of the SM resistance phenotype. Three clusters of the studied accessions were revealed. The above-mentioned resistant sources were found in two of these clusters: cluster 1—Radon, Talisman, Uralskaya 2, Zarnitsa, Talovskaya 44, Tantana, Derzhavinskaya 50, and Gran; cluster 2—Malko, Sinilga, Orlovskaya 9-2, and Pamyat Popova. The differentiation of the resistant sources into different clusters can allow the improvement of the breeding programs. The use of parental forms of different clusters in hybridization provides an opportunity to include novel variability sources in the breeding program, to obtain progeny with high genetic variability, and to enhance the target phenotype (including SM resistance) in the progeny.

In addition to the differential resistance of rye accessions to SM, we also observed the pronounced tolerance of some accessions to this disease, which was manifested in two different ways. First, some accessions (Rossianka 2, Rossul 2, and Ogonek), highly damaged by the SM at the first evaluation (one week after snow melt), showed fast regrowth and recovered well by the second evaluation (three weeks after snow melt). Second, other accessions (Carsten 2, Talovskaya 41, Hja 7009), also highly damaged by the SM, produced relatively high yields at 420–500 g/m^2^ and 240–280 g/m^2^ under NIB and AIB, respectively. This means that SM tolerance is a valuable trait for breeding directed toward the minimization of the yield losses caused by the SM.

Thus, the rye accessions analyzed in our study and the suggested options for their use in breeding are a valuable source for both traditional and genomic breeding directed toward the obtaining of SM resistant rye varieties and for elucidating the mechanisms of rye resistance and tolerance to this disease. The demand for SM resistance sources and breeding schemes to reduce SM in progenies appears to be increasing continuously, as SM not only spreads in snowy areas but also SM-causing fungi adapt well to less snowy areas and expand their geography.

## 4. Methods

### 4.1. Research Methods and Techniques

The phenotyping of winter rye genetic resources (*Secale cereale* L.) was carried out under different conditions: natural infection background (NIB) and artificially-enriched infection background (AIB), as well as using the detached leaf assay (DLA) method under controlled laboratory conditions. Experiments were performed in field trials of the Tatar Scientific Research Institute of Agriculture (a subdivision of the Federal Research Center “Kazan Scientific Center of the Russian Academy of Sciences” (KSC)), located in the center of the European part of Russia (latitude 55.649° N, longitude 49.3083° E, 60 m above sea level) during two growing seasons (2019–2020 and 2020–2021). The nurseries with NIB and AIB were located in the Laishev District of the Republic, in Bolshiye Kaban, at a distance of 1800 m from each other.

### 4.2. Climate Features

The climate of the Tatarstan area is moderately continental with cold, prolonged winters, warm, sometimes hot summers, late springs, and early autumn frosts. The coldest month of the year is January, with an average monthly temperature of −13.6 to −14.8 °C; the warmest month is July, with an average monthly temperature of +18.8 to +19.7 °C. The total annual amount of precipitation is 610 mm, with a maximum in a warm period (370–380 mm) and a minimum in a cold period (225–240 mm). The first autumn frosts are observed in the third decade of September. A stable transition of temperatures through 0 °C, to subzero temperatures, occurs at the beginning of November. Snow cover is formed in late November and remains for 145–160 days. The maximum snow cover reaches 42 cm, the average height is 30–35 cm, and the depth of ground frost is 100–120 cm. The number of frosty days per year is around 160. Spring frosts end in the second or third decade of May. A steady transition from 0 °C to positive temperatures occurs in the middle of April. Weather data was provided by the meteorological facility in Bolshiye Kaban, located near the experimental plots (Table 6).

### 4.3. Field Experiments for the Determination of SM Resistance

Screening of susceptibility to SM was carried out for genetic resources from the collection of “FRC N.I. Vavilov All-Russian Institute of Plant Genetic Resources” (VIR) and a promising gene pool of winter rye from KSC. Among 58 rye samples from 13 different countries (Russia, Belarus, Poland, Latvia, Germany, Spain, Uruguay, Argentina, China, USA, Canada, Ukraine, Finland) were tested, including cultivar Conduct—the SM susceptibility check (Supplementary Table S1).

**Figure 6:**
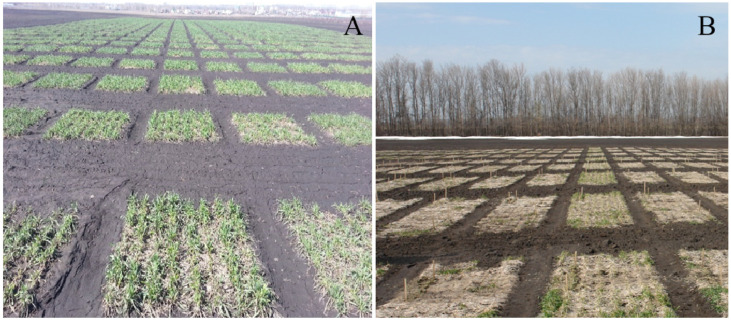
Rye plots in the nurseries with a natural infection background (NIB) (**A**) and with an artificially-enriched infection background (AIB) (**B**). The photos were taken on 15 May 2021.

Soil characteristics of both nurseries (NIB and AIB) were similar: gray forest podzolic soil (Alfisol (USDA soil taxonomy) type) with medium fertility (organic matter—3.1%, pH—6.2, nitrogen—112.0 mg/kg; phosphorus (P_2_O_5_)— 242 mg/kg; potassium (K_2_O)—56.5 mg/kg). The plants were grown in accordance with good professional practice. The field experiments (both 2019–2020 and 2020–2021) were arranged in a randomized complete block design, with two to three replications in eight-row plots of 2.5 m in length. The seeding rate was 450 kernels/m^2^. Recommended mineral fertilizer, Di-ammonium phosphate (120 kg/ha), was applied before sowing, followed by split applications of ammonium nitrate (33.5% N) of 100 kg/ha in early spring (Phenological growth stage, BBCH-identification keys of cereals scale 21). Herbicides were conventionally applied at a field location. Plots in NIB and AIB were sown at the same calendar dates, and agronomic management (field plot design, seed rate, fertilizers, and herbicides) was similar for both nurseries (Figure 6).

Since winter rye has a cross-pollinated reproductive nature, the genetically pure original seeds of each sample were used annually. To obtain a sufficient number of seeds for field and laboratory research, identical multiplication of rye samples was carried out in 1.5 m^2^ plots. Approximately 5–7 days before flowering, plants of each sample were isolated with pollen-protecting cabins (2 m high) attached to wooden tent-like frames. Cabins were made of white cotton textile, which was shaken daily (early in the morning) during flowering to improve pollination. The harvesting of plots covered with protecting cabins was done manually, and plants were threshed on a special Wintersteiger LD 180 machine.

**Figure 7:**
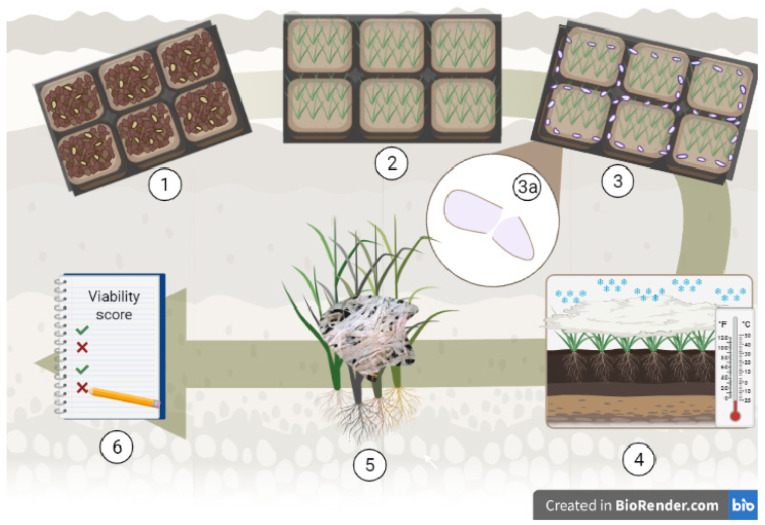
Schematic of the field experiment in an artificially-enriched infection background (AIB) nursery. (1) Sowing the plots; (2) Growing young plants up to the stage of 3 true leaves (BBCH scale 21); (3a) Soaking of the crushed grain of spring oats or barley with *Microdochium nivale*; (3) Uniform scattering of the infected grains on plots; (4) Wintering plots under snow cover, infestation of plants under snow or immediately after snow melt; (5) Visual assessment of plots as soon as the snow has melted and the field is dry enough to be able to do phenotyping of the samples (5–7 days after snow melt); (6) Disease scoring.

The AIB nursery is located inside an area protected, on three sides, by forest plantations, where snow is retained for a longer period of time, which is favorable for SM development. The AIB nursery was annually enriched with the highly virulent *M. nivale* (Fr.) Samuels & I.C. Hallett strain F00628 from the collection of KSC [46]. In 2019, the inoculum was prepared as follows: *M. nivale* was grown in potato-sucrose medium for 20 days in darkness at 20 °C. Then, the seedlings were foliar sprayed with fungal suspensions three weeks after germination. In 2020, *M. nivale* was grown under the same conditions as in 2019. Then, autoclaved crushed grains of spring oats or barley were soaked in fungal suspension for 2 h (2 mL per 100 grains) (Figure 7). The soaked grains were dried for 2 h and evenly scattered manually between the rows near the plants at the rate of 100 g of the infected grains per m^2^ when the plants reached the stage of 3 true leaves (BBCH scale 21).

There were six agronomic indicators studied in the field (at both NIB and AIB): grain yield per m^2^ (g), number of productive tillers per m^2^ (pcs), plant height (cm), winter hardiness (points), SM damage (points), and regrowth (points). Additionally, spike length (cm), spikelet number per spike (pcs), grain number per spike (pcs), grain weight per spike (g), and 1000-grain weight (g) were determined for fifteen plants of each accession under laboratory conditions.

### 4.4. Disease Scoring

Disease scoring was carried out 5–7 days after snow melt (as soon as the snow had cleared from the plot location and it was dry enough to enter the field). SM damage was scored visually for each genotype separately using the 9-point scale:

1—No damage is visible;

3—Sparse spots on lower and upper leaves (1–3 spots per leaf) with a total lesion of up to 10% of all leaves; green shoots occur on more than half of the area;

5—Lower leaves are completely affected, with 2–3 spots on the upper leaves, with a total lesion of up to 30% of all leaves; green shoots occur on less than half of the total area;

7—Lower and upper leaves are affected, lateral shoots die off, a total lesion of up to 70% of all leaves; single green shoots are visible;

9—All leaves are affected (100%), green shoots are absent due to plant death.

The even-numbered values on this scale were also used if there was good differentiation in terms of SM damage.

Based on the obtained data, the samples were classified into the following categories:

Points 1–2.9   Resistant (R)

Points 3–4.9   Moderately resistant (MR)

Points 5–6.9   Moderately susceptible (MS)

Points 7–9      Susceptible (S)

Plant regrowth was evaluated using the same visual rating scale (1–9), where “1” denotes a plant without any visible symptoms of infection and “9” was a completely dead plant with no signs of leaf elongation.

### 4.5. Detached Leaf Assay

Detached leaf assay (DLA) was carried out using a highly virulent strain of *M. nivale* F00628 [46]. DLA was performed on the leaves of 10-day-old glasshouse-grown plants, grown from the same seeds that were studied under field conditions. For each cultivar, 40 leaf segments of 3 cm in length were used. Leaves were cut off and placed in plastic containers on microscope slides; one-half of them was placed on the upper (adaxial) side of the leaf, and the second half was placed on the lower (abaxial) side of the leaf. Leaves were pressed on both sides with two layers of absorbent cotton soaked in 0.004% aqueous benzimidazole solution. The leaves were sprayed with sterile water to prevent drying. There were 5 mm-diameter plugs cut from the marginal zone of a 10-day-old *M. nivale* colony grown on potato-sucrose agar (PSA) that were placed in the middle of each leaf segment. Sterile plugs of PSA were placed on the control leave segments.

The containers were covered with glass plates and incubated in Binder chamber 720 MK (E5) (Germany) at 20 ± 1 °C with a 16 h light/8 h dark photoperiod and 60% relative humidity. Symptoms were monitored 4 days after inoculation, and the extent of disease-visible lesions in mm was assessed individually. During the laboratory screening period, 4 assessments of leaf infestation (LI), in percent of the damaged leaf area, were performed at 2-day intervals (4, 6, 8, and 10 days post infection).

As a measure of quantitative disease resistance, the area under the disease progress curve (AUDPC) was calculated according to the formula:

AUDPC = ½ (x_1_ + x_2_)(t_2_ − t_1_) + … + (x_n−1_ + x_n_)(t_n_ − t_n−1_) (1)

where AUDPC—the area under the disease progress curve; 

n—number of observations; 

x_1_, x_2_, x_n_—the percentage of visible infected area at the time of the first, second, and further registrations, %; 

t_2_ − t_1_—the time between the second and first registration, days; 

t_n_ − t_n−1_—the time between the last and next-to-last registration, days.

The index susceptibility (IS) was calculated as the ratio of AUDPC in the testing sample (AUDPC_s_) and the susceptible check (AUDPC_sc_)—cultivar Conduct.

Based on the laboratory data set, the samples were classified into the following categories:

IS 0.1–0.35     Resistant (R);

IS 0.36–0.65   Moderately resistant (MR);

IS 0.66–0.80   Moderately susceptible (MS);

IS > 0.8        Susceptible (S).

Disease incidence (DI) shows the percent of leaves with a damaged area of more than 0.5 cm. 

### 4.6. Statistical Analysis

Statistical software Microsoft Office Excel 2007 and XLSTAT 2019.2.2.59614 were used to calculate statistical characteristics and visualize the obtained data. Cluster analysis was based on the Ward’s model. The measure of distance in the agglomeration method used is the Euclidean distance. Statistically significant differences between the mean values of the analyzed features were determined (*t*-test and F-test). Results were considered significant at the probability levels of *p* < 0.05, *p* < 0.01, and *p* ≤ 0.001.

## 5. Conclusions and Future Perspectives

In our study, we identified rye SM resistance and tolerance sources and suggested hybridization schemes for breeding the varieties with improved SM resistance. We arranged a conveyer of approaches to adequately assess SM resistance and differentiate donors of genetically distinct markers of SM resistance. This is especially important for contemporary agriculture since the genetic homogeneity of crops is increasing, which ultimately leads to their increased susceptibility to infectious diseases. Although the number of molecular markers for all cereal crops, including rye, has rapidly increased in the last decade [4], they are still not available in relation to SM. Our further investigations will be devoted to the analysis of progenies obtained according to the suggested hybridization schemes, as well as the creation of the mapping populations based on the assessed plant material, in order to identify QTLs and molecular markers associated with SM resistance.

## Figures and Tables

**Table 1:** The distribution of 58 rye accessions from the genetic resources of the Vavilov All-Russian Institute of Plant Genetic Resources, by resistance categories, during the growing seasons, 2019–2020 and 2020–2021, under a natural infection background (NIB) and an artificially-enriched infection background (AIB). DS—disease score.

Resistance Level	NIB 2020	NIB 2021	AIB 2020	AIB 2021
Resistant (DS 1.0–2.9)	44	19	9	-
Moderately resistant (DS 3.0–4.9)	14	27	33	2
Moderately susceptible (DS 5.0–6.9)	-	10	12	31
Susceptible (DS 7.0–9.0)	-	2	4	25

**Table 2:** Mean ± standard deviation of disease scores determined at evaluation 1 (snow mold damage within a week after the snow melt), evaluation 2 (the plant regrowth ability two weeks after the evaluation 1), grain yield, and parameters of yield structure, for 58 rye samples, under a natural infection background (NIB) and an artificially-enriched infection background (AIB) in 2021. * Statistically significant at *p* ≤ 0.05 (*t*-test).

Trait	Mean ± Std. Deviation	
	NIB	AIB
Evaluation 1 (points)	3.52 ± 1.48 *	6.42 ± 1.17 *
Evaluation 2 (points)	3.65 ± 0.97	5.62 ± 1.18
Grain yield (g/m^2^)	416.38 ± 94.81 *	243.31 ± 72.22 *
Plant height (cm)	155.09 ± 13.13	140.55 ± 16.22
Spike length (cm)	11.09 ± 0.91	10.72 ± 0.97
Spikelet number per spike (pieces)	34.80 ± 2.31 *	29.81 ± 2.65 *
Grain number per spike (pieces)	52.18 ± 6.67	41.38 ± 8.34
Grain weight per spike (g)	1.71 ± 0.27	1.42 ± 0.33

**Table 3:** Correlation coefficients between snow mold disease scores determined 1 (disease score 1) and 3 (disease score 2) weeks after the snow melt, grain yield, and parameters of yield structure for 58 rye accessions under a natural infection background (NIB) and an artificially-enriched infection background (AIB) in 2020–2021. Values in bold are statistically significant at *p* ≤ 0.001 (*t*-test). DS—disease score.

Trait	DS 1	DS 2	Grain Yield	Plant Height	Spike Length	Spikelet Number per Spike	Grain Number per Spike	Grain Weight per Spike
NIB								
DS 1	**1**							
DS 2	**0.672**	**1**						
Grain yield	**−0.503**	**−0.574**	**1**					
Plant height	0.059	−0.149	−0.132	**1**				
Spike length	−0.215	−0.258	0.134	−0.129	**1**			
Spikelet number per spike	0.019	−0.116	0.104	−0.014	**0.626**	**1**		
Grain number per spike	0.153	−0.034	0.104	−0.127	**0.352**	**0.595**	**1**	
Grain weight per spike	−0.104	−0.084	0.233	−0.203	0.321	0.308	**0.688**	**1**
AIB								
DS 1	**1**							
DS 2	**0.551**	**1**						
Grain yield	**−0.609**	**−0.725**	**1**					
Plant height	−0.065	0.014	−0.071	**1**				
Spike length	−0.214	−0.197	0.326	−0.060	**1**			
Spikelet number per spike	−0.216	−0.170	0.328	0.076	**0.838**	**1**		
Grain number per spike	0.063	−0.096	0.195	−0.055	**0.502**	**0.653**	**1**	
Grain weight per spike	0.094	−0.013	0.167	−0.174	**0.448**	**0.575**	**0.889**	**1**

**Table 4:** Mean values of snow mold damage of rye accessions under a natural infection background (NIB) and an artificially-enriched infection background (AIB) in the field, as well as index susceptibility (IS), mean percent of leaf infestation (LI), and mean percent of disease incidence (DI), determined using a detached leaf assay following *Microdochium nivale* infection.

Name	AIB, Points	NIB, Points	IS	LI, %	DI, %
Radon	4.0	1.7	0.47	16.3	63.3
Falenskaya 4	4.0	2.8	0.73	25.4	86.7
Talisman	4.0	1.8	0.52	17.7	80.9
Derzhavinskaya 50	4.3	3.0	0.63	21.3	77.5
Uralskaya 2	4.3	2.5	0.58	20.0	71.7
Tantana	4.5	1.5	0.65	22.0	70.8
Talovskaya 2	4.5	1.8	1.03	35.2	75.0
Gran	4.5	3.8	0.50	17.3	75.8
Talovskaya 44	4.5	3.0	0.56	19.2	70.8
Zarnitsa	4.5	2.0	0.43	15.0	62.5
Malko	4.5	1.8	0.52	17.8	57.5
Rifle Fall	4.5	2.5	1.48	50.6	96.7
Rossul 2	4.8	2.5	1.09	36.8	89.2
Sinilga	4.8	2.3	0.23	7.9	35.9
Pamyat Popova	4.8	2.5	0.28	9.7	36.7
Orlovskaya 9-2	4.8	3.0	0.49	16.9	53.4
Chishminskaya 3-2	5.0	2.5	0.90	30.4	85.0
Krona 2	5.0	2.8	1.56	53.9	96.7
Adar	5.0	4.3	0.45	15.5	65.0
Toseuschi	5.0	2.5	0.66	23.3	87.5
Roxana	5.3	2.3	0.47	15.7	69.2
Carsten 2	5.3	3.8	0.38	13.1	46.7
Olga	5.5	3.3	0.34	11.7	51.7
Slavia	5.5	1.8	0.51	17.2	61.7
Ogonek	5.8	2.8	0.14	4.6	25.8
Tatiana	6.0	3.0	0.49	16.8	44.2
Parcha	6.0	2.8	1.17	40.7	87.5
Zduno	6.0	3.5	1.18	40.5	83.3
Yaseldya	6.5	3.5	0.34	11.6	50.8
Kaupo	6.5	2.5	0.57	20.9	50.0
Marusenka	7.0	3.0	0.28	10.2	39.2
Solnyshko	7.0	3.0	0.96	33.8	84.2
Jan An	7.3	4.5	0.51	17.3	49.2
Conduct	8.8	4.5	1.00	34.8	85.0

**Table 5:** The characteristics of clusters arranged based on the parameters of snow mold damage of 34 rye accessions assessed under a natural infection background (NIB) and an artificially-enriched infection background (AIB) in the field, as well as under laboratory conditions following *Microdochium nivale* infection using a detached leaf assay yielding index susceptibility (IS), mean percent of leaf infestation (LI), and mean percent of disease incidence (DI).

Cluster	Number of Accessions	Name	AIB, Points	NIB, Points	IS	LI, %	DI, %
1	13	Tantana, Radon, Falenskaya 4, Derzhavinskaya 50, Uralskaya 2, Roxana, Gran, Slavia, Talovskaya 44, Zarnitsa, Talisman, Adar, Toseuschi	4.6	2.5	0.6	18.9	72.6
2	12	Ogonek, Tatiana, Olga, Sinilga, Marusenka, Carsten 2, Pamyat Popova, Orlovskaya 9-2, Yaseldya, Malko, Kaupo, Jan An	5.7	3.0	0.4	13.2	45.1
3	9	Chishminskaya 3-2, Rossul 2, Krona 2, Talovskaya 2, Solnyshko, Parcha, Zduno, Conduct, Rifle Fall	5.7	2.9	1.2	39.6	87.0

**Table 6:** Winter weather conditions provided by the meteorological facility in Bolshiye Kaban, 2019–2021.

Meteorological Parameters	2019–2020	2020–2021
Start of winter	19 Nov	11 Nov
The end of winter	8 Mar	26 Mar
Length of winter, days	110	135
Duration of snow cover, days	102	150
Number of days with temperature −20…−30 °C	1	1
Minimal air temperature for the winter period, °C	−20	−31
Number of thaws for the winter period, days	61	12
Average air temperature for the winter period, °C	−2.4	−8.6
Amount of precipitation, mm	135	172

## Data Availability

Not applicable.

## Supplementary Materials

The following supporting information can be downloaded at: https://www.mdpi.com/article/10.3390/plants11192516/s1, Table S1: The genetic resources from the collection of “FRC N.I. Vavilov All-Russian Institute of Plant Genetic Resources” (VIR) and a promising gene pool of winter rye from KSC tested in the present study; Table S2: SM disease scores of 58 rye accessions from the genetic resources of VIR assessed during the growing seasons 2019–2020 and 2020–2021 under a natural infection background (NIB) and an artificially-enriched infection background (AIB); Table S3: Disease incidence (DI), leaf infestation (LI) at 4, 6, 8, and 10 days post infection (1–4 score), and index susceptibility (IS) assessed by the detached leaf assay (DLA) on leaf segments of 34 rye accessions inoculated with *Microdochium nivale*.
